# Recent advances in understanding the physiology of hypoxic sensing by the carotid body

**DOI:** 10.12688/f1000research.16247.1

**Published:** 2018-12-06

**Authors:** Nanduri R. Prabhakar, Ying-Jie Peng, Jayasri Nanduri

**Affiliations:** 1 Institute for Integrative Physiology and Center for Systems Biology of O2 Sensing, The University of Chicago, Chicago, IL, 60637, USA

**Keywords:** Carotid body, Heme-oxygenase, Gasotransmitter, NADH dehydrogenase Fe-S protein 2

## Abstract

Hypoxia resulting from reduced oxygen (O _2_) levels in the arterial blood is sensed by the carotid body (CB) and triggers reflex stimulation of breathing and blood pressure to maintain homeostasis. Studies in the past five years provided novel insights into the roles of heme oxygenase-2 (HO-2), a carbon monoxide (CO)-producing enzyme, and NADH dehydrogenase Fe-S protein 2, a subunit of the mitochondrial complex I, in hypoxic sensing by the CB. HO-2 is expressed in type I cells, the primary O2-sensing cells of the CB, and binds to O _2_ with low affinity. O _2_-dependent CO production from HO-2 mediates hypoxic response of the CB by regulating H _2_S generation. Mice lacking NDUFS2 show that complex I-generated reactive oxygen species acting on K ^+^ channels confer type I cell response to hypoxia. Whether these signaling pathways operate synergistically or independently remains to be studied.

## Introduction

Systemic hypoxia, which arises from decreased oxygen (O _2_) levels in the arterial blood, is a fundamental physiological stimulus. The duration of hypoxia can be acute, ranging from seconds to minutes, or chronic, lasting hours to days. Acute hypoxia evokes rapid changes in the cardiorespiratory systems to ensure optimal O _2_ delivery to tissues. Cardiorespiratory responses to acute hypoxia are primarily reflexive in nature, initiated by sensory organs located in the carotid artery and aorta. Carotid bodies (CBs), which reside at the bifurcation of the common carotid arteries, are the major sensory organs for monitoring arterial blood O _2_ levels ^1^. Although structures similar to CBs are seen at the aortic arch and in the abdominal arteries, much of the information on the mechanisms of hypoxic sensing has come from studies on the CB ^1^. Here, we present studies reported in the past five years on the roles for heme oxygenase-2 (HO-2), a carbon monoxide (CO)-synthesizing enzyme, and NDUFS2, a mitochondrial complex I subunit, in hypoxic sensing by the CB.

## Physiology of hypoxic sensing by the carotid body

The CB receives sensory innervation from the carotid sinus nerve, whose cell bodies reside in the petrosal ganglion. Under basal conditions (arterial blood pO _2_ of about 100 mmHg), sensory nerve discharge (that is, frequency of action potentials) is low. In response to even a modest decrease in arterial blood pO _2_ from 100 to 80 mmHg, the sensory discharge increases and the response is fast, occurring within a few seconds after the onset of hypoxia ^1^. The increased sensory discharge is non-adapting and is maintained during the entire duration of hypoxia ^2^ or may progressively increase during sustained hypoxia, lasting several hours ^3^. The exquisite sensitivity and the speed of the response with little or no adaptation are the unique features of hypoxic sensing by the CB. The increased CB sensory nerve activity is relayed to brainstem neurons, leading to reflex stimulation of breathing and blood pressure (CB chemo reflex) ^1^.

The CB tissue is made of two major cell types: type I cells (also called glomus cells), which are of neuronal origin, and type II cells, which resemble glial cells of the nervous system. Type I cells along with the nearby sensory nerve ending function as a “sensory unit” ^1^. Stimulus response of breathing to graded hypoxia parallels the CB sensory nerve activity ^1^. Consequently, carotid sinus nerve activity is measured as an index of CB hypoxic sensing ^1^. Type I cell responses to acute hypoxia are measured by monitoring exocytosis and changes in [Ca ^2+^] _i_ and K ^+ ^channel conductance ^1^. Although type I cells respond to hypoxia with elevated [Ca ^2+^] _i_ or K ^+^ channel inhibition (or both), they are not always reflected in the sensory nerve activity ^4, 5^, which is essential for evoking the physiologically important CB chemo reflex. Therefore, it is necessary to corroborate the cellular responses to hypoxia with the sensory nerve discharge for assessing the physiological relevance of CB hypoxic sensing.

## Transduction mechanisms

The consensus is that hypoxia inhibits certain K ^+^ channels in type I cells and the resulting depolarization leads to Ca ^2+^-dependent release of neurotransmitter or neurotransmitters, which stimulate the nearby sensory nerve ending, leading to increased sensory discharge ^1^. The roles for K ^+^ channels and AMP kinase (AMPK) in CB hypoxic sensing have been discussed in detail elsewhere ^1, 6, 7^ and will not be presented in this commentary. The following section presents studies conducted in the past five years that have provided novel insights into the roles for HO-2 and mitochondrial complex subunit NDUFS2 in hypoxic sensing by the CB.

## Heme oxygenase-2

Type I cells express HO-2-like immunoreactivity ^8^. HO-2 is remarkably sensitive to O _2_ availability, and graded hypoxia progressively decreases CO production in the CB ^9^. Reduced CO production by hypoxia is also seen in HEK-293 cells with heterologous expression of HO-2 ^9^, suggesting that HO-2 is inherently sensitive to O _2_. HO-2 binds to O _2_ with low affinity with an apparent K _m_ of 65 ± 5 mmHg (about 80 µM). The O _2_ sensitivity of HO-2 is due to Cys ^265^ and Cys ^282^ residues in the heme regulatory motif ^9^. Intact Cys ^265^ and Cys ^282^ residues lower the affinity of HO-2 for O _2_ and thereby enable the enzyme to transduce changes in O _2_ into changes in CO production. Substituting Cys ^265^ and Cys ^282^ with alanine allows the HO-2 to bind to O _2_ with high affinity ^9^.

**Figure 1.**
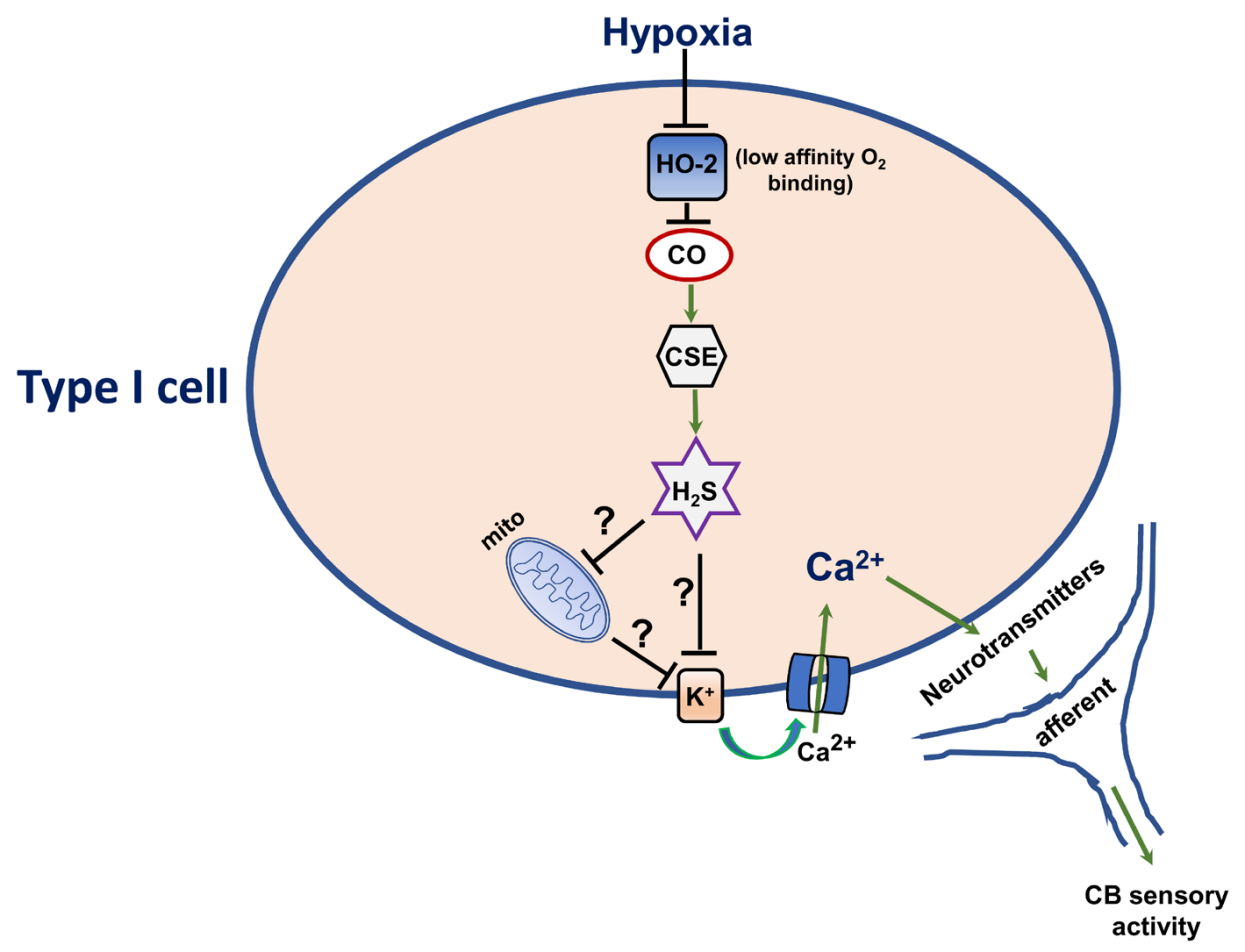
Heme oxygenase-2 (HO-2) signaling in hypoxic sensing by the carotid body (CB). Ca ^2+^, calcium channel; CO, carbon monoxide; CSE, cystathionine gamma-lyase; H _2_S, hydrogen sulfide; K ^+^, potassium channel; mito, mitochondria.

It was proposed, based on the findings that CO inhibits CB sensory nerve excitation by hypoxia ^8– 10^ and that hypoxia reduces CO production in a stimulus-dependent way ^9^, that low sensory nerve activity during normoxia is due to high CO levels inhibiting CB sensory nerve activity but that hypoxia, by reducing CO production, relieves the inhibition and thereby increases the sensory nerve discharge ^8^. Recent studies determined how CO inhibits CB sensory nerve activity under normoxia ^9, 10^. Type I cells also express cystathionine gamma-lyase (CSE), an enzyme catalyzing hydrogen sulfide (H _2_S) production ^11^. H _2_S is a potent stimulator of CB sensory nerve activity in rats, mice, rabbits, and cats ^11– 13^. During normoxia, H _2_S levels are low and hypoxia increases H _2_S levels in a stimulus-dependent manner ^11^. The increased H _2_S production is not due to inherent O _2_ sensitivity of CSE; rather, it is due to changes in CO production ^9^. High CO levels during normoxia inhibit CSE-derived H _2_S generation through protein kinase G (PKG)-dependent phosphorylation of Ser ^377^ of CSE, and reduced CO generation during hypoxia relieves the inhibition of CSE, leading to increased H _2_S generation in the CB ^9^. CSE inhibitors and CSE knockout mice exhibit impaired type I cell and sensory nerve and breathing response to hypoxia ^11, 14^. These findings suggest that low sensory discharge during normoxia is due to inhibition of H _2_S generation by high levels of HO-2-derived CO but that the increased sensory nerve activity by hypoxia is due to relieving inhibition of H _2_S synthesis by CO ( Figure 1).

Genetic disruption of HO-2 increases baseline CB sensory nerve activity and elevates H _2_S levels under normoxia, a phenotype similar to hypoxia ^9^. However, hypoxia further increased CB sensory activity and elevated H _2_S levels in HO-2 null CBs, indicating the existence of a redundant hypoxic sensing mechanism (or mechanisms). This redundant hypoxic sensing was due to compensatory upregulation of neuronal nitric oxide synthase (nNOS) in type I cells of HO-2 null mice ^9^. nNOS, like HO-2, also binds to O _2_ with low affinity, and nitric oxide (a product of nNOS), like CO, also inhibits CSE-derived H _2_S through PKG signaling ^9^. Blockade of nNOS in HO-2 null mice renders CBs completely insensitive to hypoxia ^9^. These findings suggest that, in the absence of HO-2, nNOS contributes to CB hypoxic sensing by regulating CSE-derived H _2_S production ^9^.

A recent study examined the role for HO-2–CO signaling in CB hypoxic sensing of three genetically distinct rat strains that are commonly used in experimental research ^10^. As compared with Sprague-Dawley rat CB, Brown-Norway (BN) rat CB showed markedly attenuated sensory nerve and type I cell responses to hypoxia, and this phenotype was associated with higher CO and lower H _2_S levels in the glomus tissue. The elevated CO levels in the BN rat CB were due to high affinity of HO-2 to its substrate hemin ^10^. The attenuated CB response to hypoxia is associated with a blunted chemo reflex. The CB chemo reflex is essential for ventilatory adaptations to high-altitude hypoxia ^15, 16^. BN rats exposed to hypobaric hypoxia showed severe pulmonary edema ^10^, which is a sign of chronic mountain sickness. Treating BN rats with a HO inhibitor restored the hypoxic response of the CB and prevented pulmonary edema caused by hypobaric hypoxia ^10^.

In contrast to BN rat CBs, CBs of spontaneous hypertensive (SH) rats showed heightened sensitivity to hypoxia, and this phenotype is associated with low CO levels and high H _2_S levels in the CB ^10^. The low CO levels in SH rat CBs were due to low hemin affinity of HO-2 ^10^. Current evidence suggests that a hyperactive CB chemo reflex is a major contributor of hypertension in SH rats ^17, 18^. Systemic administration of a CSE inhibitor normalized CB sensory nerve and type I cell responses to hypoxia and reduced hypertension ^10^. Although chronic ablation of CB also reduced hypertension to the same level as seen with a CSE inhibitor, combined CB ablation and CSE inhibitor treatment had no further effect on blood pressure ^10^. These findings suggest that CO-regulated H _2_S contributes to a hyperactive CB in SH rats. It has long been known that the CB chemo reflex exhibits substantial inter-individual variations in humans and experimental animals ^19– 21^. Studies on BN and SH rats indicate that inter-individual variations in chemo reflex are due in part to variations in HO-2–CO signaling in the CB.

A recent study suggests that HO-2–CO signaling in the CB also plays an important role in the pathology of sleep apnea (SA), which is a highly prevalent respiratory disease ^22^. SA is characterized by episodic cessation of breathing leading to chronic intermittent hypoxia (CIH). Patients with SA and CIH-exposed rodents exhibit a heightened CB chemo reflex, leading to chronic elevation of sympathetic nerve activity and hypertension ^23– 26^. Rodents exposed to CIH exhibit elevated reactive oxygen species (ROS) levels in the CB ^27, 28^. Peng *et al*. showed that ROS inhibit CO generation by HO-2 by acting on the Cys ^265^ residue in the heme regulatory motif, thereby increasing H _2_S levels in the CB ^22^. Pharmacological or genetic blockade of CSE-derived H _2_S prevents CIH-induced CB hyperactivity, sympathetic nerve excitation, and hypertension ^22^. Collectively, these studies suggest that disrupted HO-2–CO signaling in the CB leads to dire physiological consequences.

Although the studies described above suggest that CO-regulated H _2_S is an important mediator of CB hypoxic sensing, a recent study questioned this possibility. Kim *et al*. ^29^ reported that inhibitors of H _2_S synthesis had no effect on [Ca ^2+^] _i_ and TASK K ^+^ channel responses of type I cells to anoxia (pO _2_ of about 5 mmHg). Peng *et al*. ^5^ re-examined the role for CSE-derived H _2_S in the CB sensory nerve and type I cell [Ca ^2+^] _i_ responses to hypoxia (pO _2_ of about 37 mmHg) and anoxia (pO _2_ of about 5 mmHg). The authors found that hypoxia increased H _2_S levels in the CB, stimulated sensory nerve activity, and elevated [Ca ^2+^] _i_ in type I cells and all of these responses were blocked by a CSE inhibitor and in CSE knockout mice. In striking contrast, anoxia, though producing very low pO _2_, had no effect on H _2_S levels in the CB and produced only a weak CB sensory nerve excitation as compared with hypoxia. CB sensory and type I cell responses to anoxia were unaffected by CSE inhibitors and in CSE knockout mice. Moreover, anoxia (100% N _2_) depressed breathing whereas hypoxia (12% O _2_) stimulated breathing ^5^. CSE knockout mice showed an absence of breathing stimulation by hypoxia, whereas the depressed breathing by anoxia was unaffected in these mice ^5^. These findings suggest that hypoxia and anoxia are not the same stimuli for studying CB physiology and that HO-2–CO-regulated H _2_S mediates CB response to “physiologically relevant” hypoxia but not anoxia.

## NADH dehydrogenase Fe-S protein 2 (NDUFS2) mitochondrial complex I subunit

Mitochondrial electron transport chain (ETC) inhibitors mimic the effects of hypoxia on CB sensory nerve activity ^1^ and type I cells ^30– 33^. Mills and Jöbsis ^34^ reported that CBs express a putative cytochrome aa3 with two O _2_ affinities: one with high and another with low affinity for O _2_. Based on spectral analysis, subsequent studies suggested that CBs express a cytochrome, which is half-reduced at a pO _2_ of 60 to 80 mmHg, and this cytochrome is not expressed in either the superior cervical or the nodose ganglion ^35, 36^. Acute hypoxia increased the NADH/NAD ratio and decreased mitochondrial membrane potential in type I cells, and these effects were not seen in other non-chemoreceptor tissues such as dorsal root ganglion ^37^. These studies led to the suggestion that mitochondrial ETC participates in CB hypoxic sensing.

Rotenone, an inhibitor of the mitochondrial complex I, selectively blocks the type I cell response to hypoxia ^32^. NDUFS2 is a component of the complex I, which binds to ubiquinone ^38– 40^. Recent studies examined the role for complex I in CB hypoxic sensing in mice with targeted deletion of *Ndufs2* in tyrosine hydroxylase-positive (TH ^+^) cells such as type I cells ^41^. Mice lacking NDUFS2 in TH ^+^ cells showed an absence of breathing stimulation by hypoxia and of hypoxia-evoked exocytosis and K ^+^ channel inhibition in type I cells. However, type I cell responses to severe hypercapnia (20% CO _2_) were intact ^41^. Given that these mice have a deletion of NDUFS2 since birth, the lack of breathing response to hypoxia might be secondary to metabolic adaptations during development. Additional studies were performed on adult (2-month-old) mice with conditional knockout of *Ndufs2* (ESR-NDUFS2 mice). Like the TH-NDUFS2 mice, mice with conditional knockdown of *Ndufs2* showed an absence of stimulation of breathing as well as type I cell responses to hypoxia ^41^. The lack of cellular responses to hypoxia was associated with decreased complex I activity, complex I formation, and complex I-dependent O _2_ consumption, whereas the functions of other mitochondrial complexes were intact. These studies suggest that NDUFS2 of the mitochondrial complex I contributes to hypoxic sensing by the CB.

**Figure 2.**
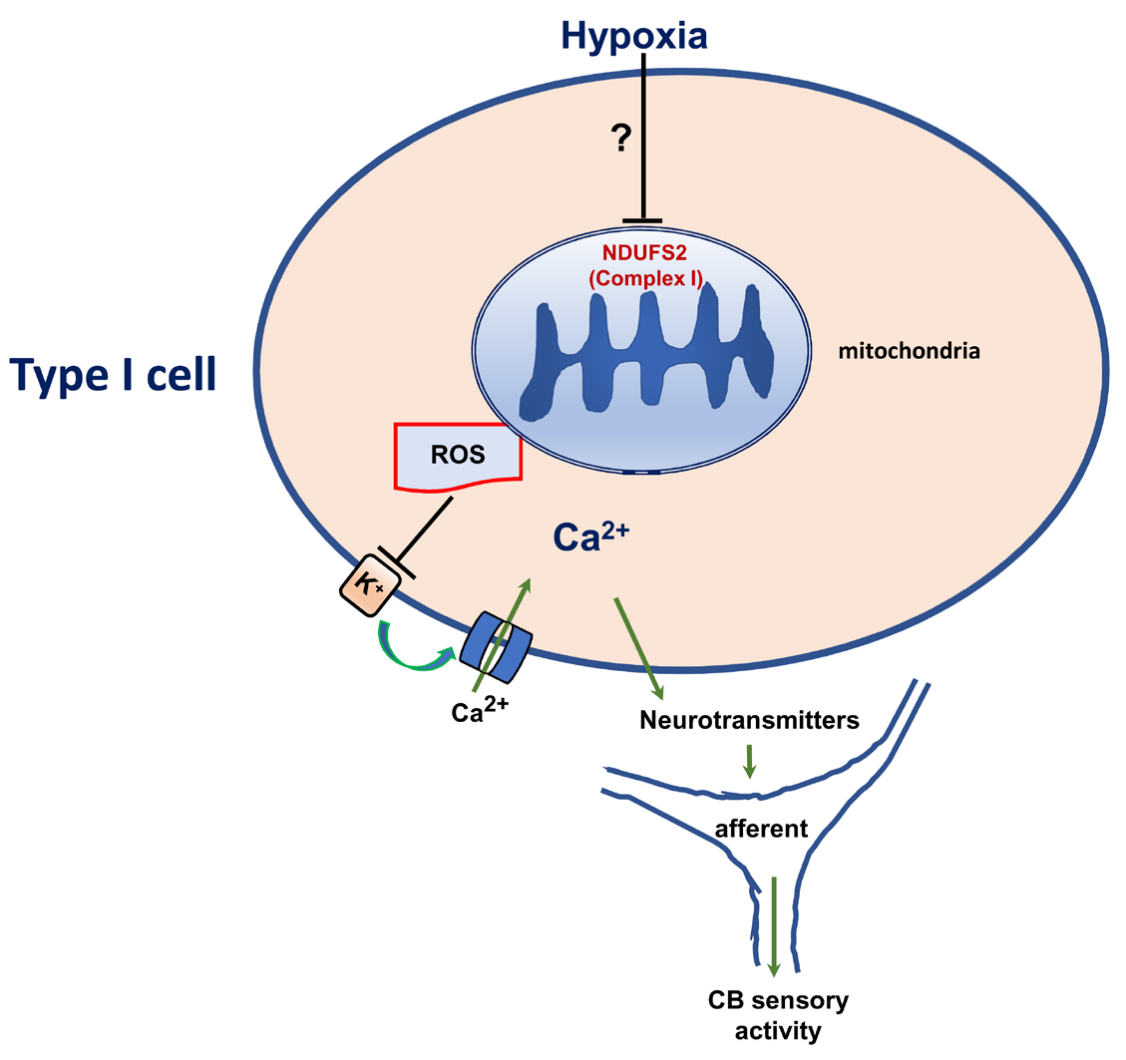
NADH dehydrogenase Fe-S protein 2 (NDUFS2), a mitochondrial complex I subunit, signaling in hypoxic sensing by the carotid body (CB). Ca ^2+^, calcium channel; K ^+^, potassium channel; ROS, reactive oxygen species.

How might NDUFS2 confer hypoxic sensitivity on type I cells? Mitochondrial ETC-generated ROS have been implicated in pulmonary artery myocyte responses to acute hypoxia ^42– 44^. Acute hypoxia increased ROS in wild-type type I cell cytosol and mitochondrial intermembrane space, and these responses were attenuated in NDUFS2 null type I cells ^41^. Intracellular application of H _2_O _2_, like hypoxia, inhibited background K ^+^ currents in type I cells ^41^. These findings led to the suggestion that inhibition of NDUFS2 leads to an increase in ROS production, which in turn (by inhibiting K ^+^ currents) leads to depolarization of type I cells by hypoxia ( Figure 2).

## Summary and future directions

It has long been thought that an O _2 _sensor or sensors in type I cells initiate hypoxic sensing in the CB ^1, 45, 46^. To be considered an O _2_ sensor, a molecule should satisfy certain criteria, namely (a) its presence in type I cells, (b) low-affinity binding to O _2_, (c) altered function by hypoxia should initiate signaling events leading to increased CB sensory nerve activity, and (d) loss of CB hypoxic sensing by disrupting its function. HO-2 satisfies the proposed criteria for an O _2_ sensor in the CB and contributes to CB sensory excitation by regulating H _2_S production through O _2_-dependent CO production. However, further studies are needed to delineate the cellular mechanism(s) underlying CB activation by H _2_S. H _2_S donors, like hypoxia, depolarize ^47^ and inhibit K ^+^ channel conductance in type I cells ^12, 48^ and increase NADH auto-fluorescence in type I cells, an effect attributed to the inhibition of mitochondrial ETC ^47^. It is likely that H _2_S mediates sensory nerve excitation by hypoxia by inhibiting mitochondrial ETC, thereby affecting K ^+^ conductance of type I cells ( Figure 1).

Studies with genetically engineered mice suggest that the inactivation of NDUFS2 is an important step for the type I cell response to hypoxia. However, it remains to be determined whether graded hypoxia inhibits NDUFS2 and establishes the affinity of O _2_ for this enzyme. NDUFS2 is a ubiquitously expressed enzyme in the body. However, unlike many other tissues, CBs are highly sensitive to changes in O _2_ levels. Consequently, the uniqueness of NDUFS2 signaling in the CB remains to be established.

Finally, whether HO-2 and NDUFS2 signaling operates independently or works in concert is not clear. The CB responds to a wide range of pO _2_ values (about 80–20 mmHg). It was proposed that interactions between multiple signaling pathways working in concert as a “chemosome” enable the CB to sense a wide range of pO _2_ values ^45, 46^. Given that high concentrations of H _2_S can inhibit mitochondrial ETC ^47^, HO-2 and NDUFS2 signaling might work in concert as a “chemosome” for the full expression of the CB to a wide range of hypoxia, a possibility that remains to be investigated.
